# Real-Time Artificial Intelligence-Based Histologic Classifications of Colorectal Polyps Using Narrow-Band Imaging

**DOI:** 10.3389/fonc.2022.879239

**Published:** 2022-04-26

**Authors:** Yi Lu, Jiachuan Wu, Xianhua Zhuo, Minhui Hu, Yongpeng Chen, Yuxuan Luo, Yue Feng, Min Zhi, Chujun Li, Jiachen Sun

**Affiliations:** ^1^Department of Gastrointestinal Endoscopy, the Sixth Affiliated Hospital, Sun Yat-sen University, Guangzhou, China; ^2^Guangdong Provincial Key Laboratory of Colorectal and Pelvic Floor Diseases , the Sixth Affiliated Hospital, Sun Yat-sen University, Guangzhou, China; ^3^Digestive Endoscopy Center, Guangdong Second Provincial General Hospital, Guangzhou, China; ^4^Department of Otorhinolaryngology, the Second Affiliated Hospital, Sun Yat-sen University, Guangzhou, China; ^5^Tianjin Economic-Technological Development Area (TEDA) Yujin Digestive Health Industry Research Institute, Tianjin, China; ^6^Department of Gastroenterology, the Sixth Affiliated Hospital, Sun Yat-sen University, Guangzhou, China

**Keywords:** artificial intelligence, colorectal, polyp, NBI, NICE

## Abstract

**Background and Aims:**

With the development of artificial intelligence (AI), we have become capable of applying real-time computer-aided detection (CAD) in clinical practice. Our aim is to develop an AI-based CAD-N and optimize its diagnostic performance with narrow-band imaging (NBI) images.

**Methods:**

We developed the CAD-N model with ResNeSt using NBI images for real-time assessment of the histopathology of colorectal polyps (type 1, hyperplastic or inflammatory polyps; type 2, adenomatous polyps, intramucosal cancer, or superficial submucosal invasive cancer; type 3, deep submucosal invasive cancer; and type 4, normal mucosa). We also collected 116 consecutive polyp videos to validate the accuracy of the CAD-N.

**Results:**

A total of 10,573 images (7,032 images from 650 polyps and 3,541 normal mucous membrane images) from 478 patients were finally chosen for analysis. The sensitivity, specificity, PPV, NPV, and accuracy for each type of the CAD-N in the test set were 89.86%, 97.88%, 93.13%, 96.79%, and 95.93% for type 1; 93.91%, 95.49%, 91.80%, 96.69%, and 94.94% for type 2; 90.21%, 99.29%, 90.21%, 99.29%, and 98.68% for type 3; and 94.86%, 97.28%, 94.73%, 97.35%, and 96.45% for type 4, respectively. The overall accuracy was 93%. We also built models for polyps ≤5 mm, and the sensitivity, specificity, PPV, NPV, and accuracy for them were 96.81%, 94.08%, 95%, 95.97%, and 95.59%, respectively. Video validation results showed that the sensitivity, specificity, and accuracy of the CAD-N were 84.62%, 86.27%, and 85.34%, respectively.

**Conclusions:**

We have developed real-time AI-based histologic classifications of colorectal polyps using NBI images with good accuracy, which may help in clinical management and documentation of optical histology results.

## Introduction

Colorectal cancer ranks as one of the most common cancers and the second leading cause of cancer death worldwide (1). Colonoscopy screening is of vital importance to the prevention of colorectal cancer. In colonoscopy screening, precancerous polyps, such as adenomas and serrated polyps, are resected. In recent years, with the development of convoluted neural networks (CNNs) or artificial intelligence (AI), we have become capable of applying real-time computer-aided detection (CAD) in clinical practice (2, 3), and these AI-based polyp detection systems are reported to increase the rate of polyp detection (4). However, a certain portion of these polyps are hyperplastic polyps, and resection of these polyps can increase medical costs, and sometimes we take a lot of time to search for diminutive polyps that tend out to be hyperplastic polyps. On the one hand, as malignant transformations of these polyps are rare, a pathologic evaluation might be unnecessary. On the other hand, some patients with early colorectal cancer diagnosed after surgical resection might have been cured by less invasive endotherapy if we knew their histopathology result before surgery. Therefore, accurate histologic diagnosis before endoscopic resection has the potential to improve the cost-effectiveness and efficiency of colonoscopy, preventing unnecessary endotherapy and relieving the financial burden (5).

Narrow-band imaging (NBI), a kind of image-enhanced technology, helps us visualize the surface patterns and vessels of polyps, and it has been reported to provide valuable information regarding the histology of polyps (6). The NBI International Colorectal Endoscopic (NICE) classification is a simplified diagnostic criterion for the diagnosis of colorectal polyp histology and is widely used in centers without magnifying endoscopy (7, 8). However, due to its subjective nature, interobserver agreement varies in different situations, especially for non-experts (9, 10). To address this problem, previous studies successfully developed AI-based CADx to differentiate hyperplastic and neoplastic polyps with NBI images (5, 11), but they did not characterize deep submucosal invasive carcinoma (NICE classification type 3). Later, Okamoto et al. developed a CADx-N model to differentiate the 3 types of NICE classification. The overall accuracy was excellent, but the specificity and sensitivity for some types were not that satisfactory, as the specificity for NICE classification type 2 (low-grade dysplasia to submucosal-shallow invasive carcinoma) was 80.6%, and the sensitivity for NICE classification type 3 was 61.1% (12). This CADx was developed in Japan, and its utilization in China has a long way to go. Moreover, the method used for developing their CADx was Residual Network (ResNet), and there is a new variant of it, preserving its structure without introducing additional computational costs and with better performance, which was named ResNeSt (13). Therefore, we used more NBI images to develop an AI-based CAD-N with ResNeSt to make real-time histologic classifications of colorectal polyps with good accuracy and validated its accuracy with videos.

## Methods

### Clinical Data and Polyp Image Collection

We retrospectively searched for colorectal NBI images at the Sixth Affiliated Hospital, Sun Yat-sen University, and Guangdong Second Provincial Central Hospital. These images were used to train the model and have internal validation tests. The scopes used in this study were PCF-Q260JI, CF-H260AI, PCF-Q260AZI, PCF-Q260A3I, CF-H290I, CF-HQ290I, PCF-H290ZI, and CF-HQ290Z (Olympus Optical Co., Ltd., Tokyo, Japan), and the machines we used were CV-260 and CV-290 (Olympus Medical Systems). The collected images met the following criteria: NBI images; with pathology (if the image was normal mucosa, then pathology was not necessary); the pathology was made by the whole resection specimens rather than biopsy; good quality; and repeated or blurred images were excluded. Additionally, we collected the following information of the patients: age, sex, and information of the polyps: location, size, number, and pathology. The collected images were categorized into 4 types according to their pathology: type 1, hyperplastic or inflammatory polyps; type 2, adenomatous polyps, intramucosal cancer, or superficial submucosal invasive cancer; type 3, deep submucosal invasive cancer; and type 4, normal mucosa.

### Development of CAD-N

A kind of deep convolutional neural network (DCNN) classifier named ResNeSt was used to train the model (13). The process was mainly implemented with Python (Version 3.7) and PyTorch (Version 1.7.1). The development of CAD-N was supported by TEDA Yujin Digestive Health Industry Research Institute.

**Figure 1:**
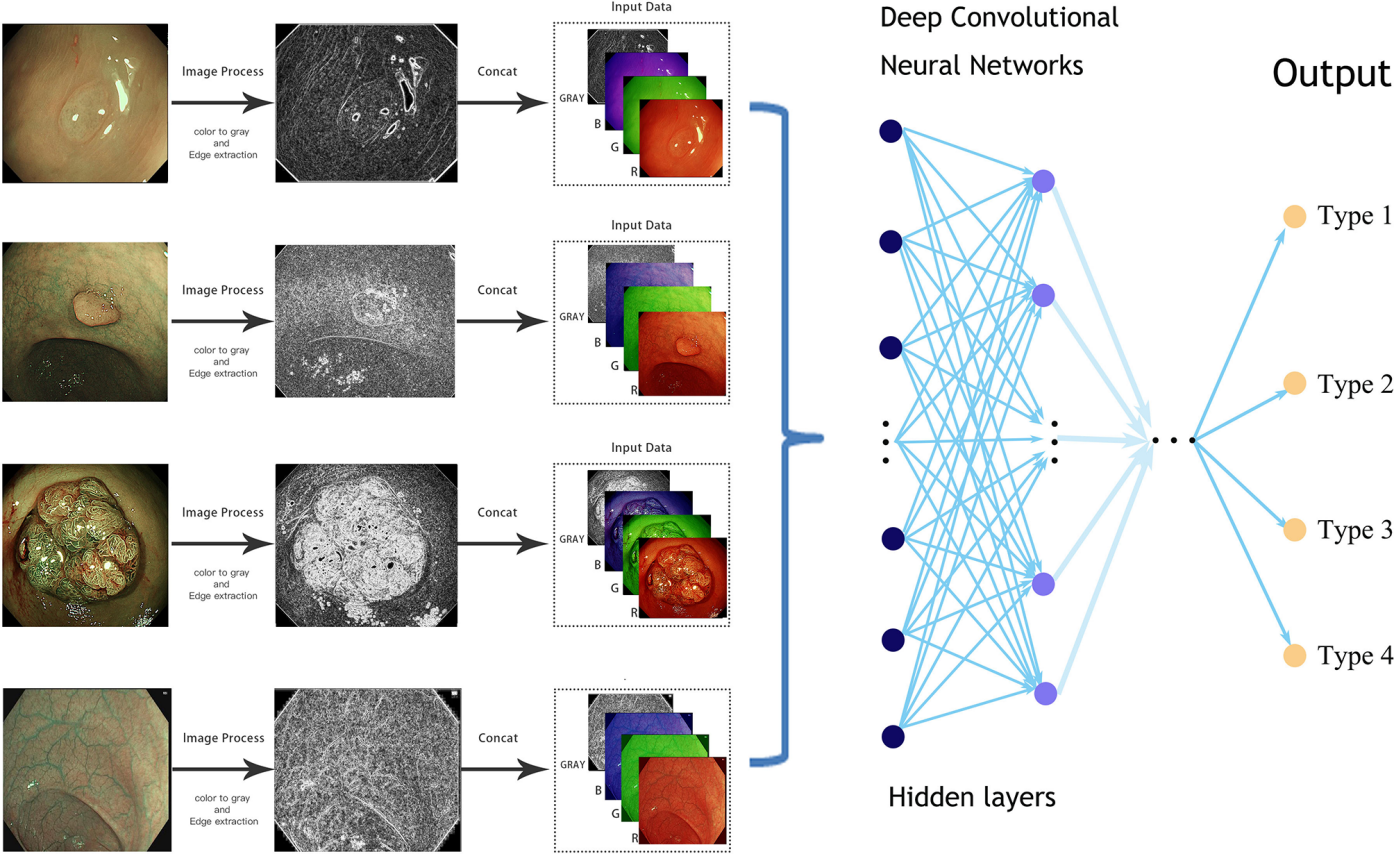
Simplified schematic diagram of the deep learning methods from an endoscopic image to the final prediction.

**Figure 2:**
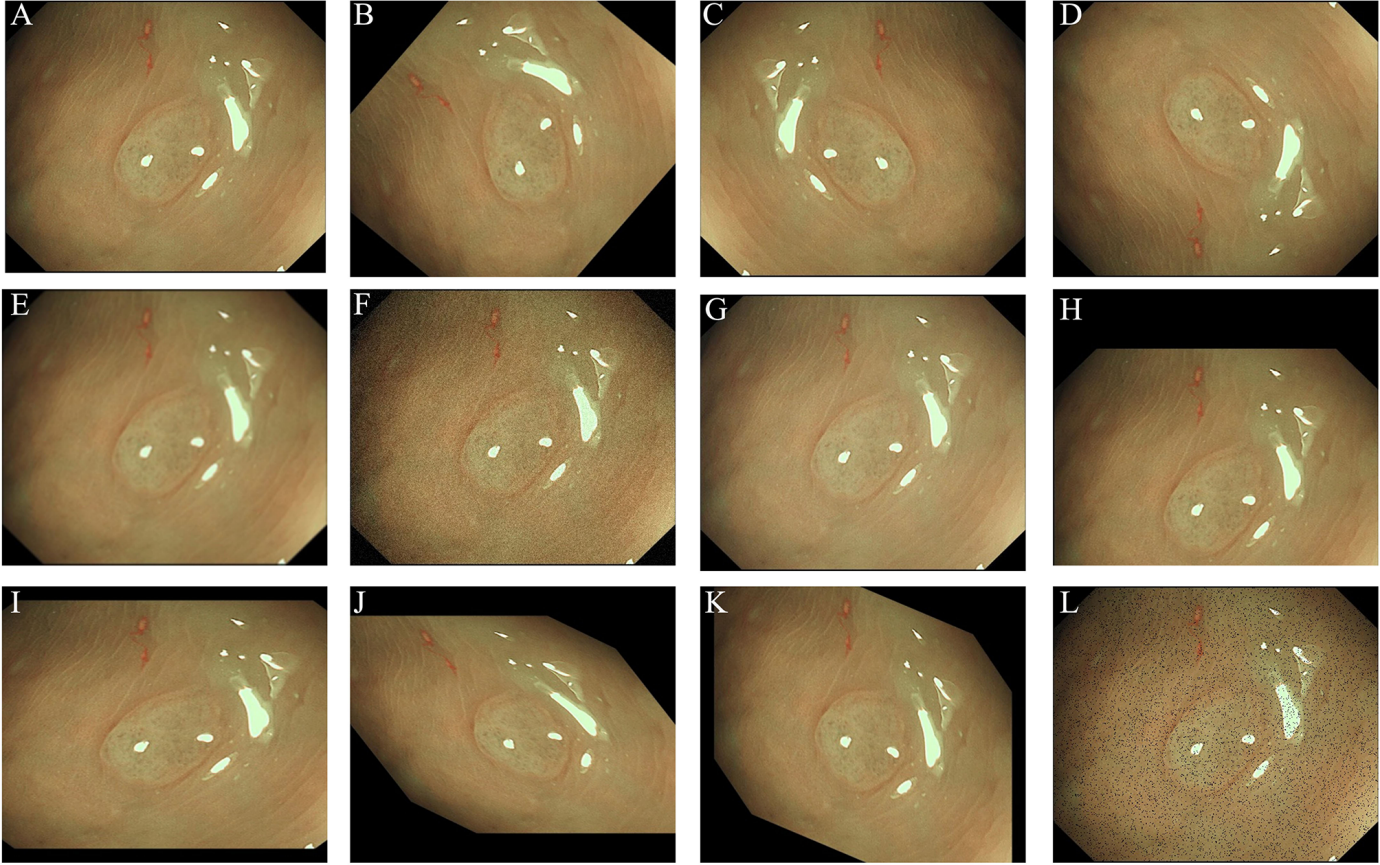
Images showing the data argumentation methods used in this study: **(A)** original image; **(B)** rotation; **(C)** horizontal flipping; **(D)** vertical flipping; **(E)** image blur; **(F)** random noise; **(G)** gamma contrast variation; **(H)** random translation; **(I)** random scaling; **(J)** inclination (left and right); **(K)** inclination (up and down); **(L)** random discard pixels.

As in the NBI images, the surface patterns and vessels of polyps are important elements to distinguish their pathology, so edge gradient extraction was adopted for each NBI image by using OpenCV (Version 4.2.0). The obtained gray edge gradient image channel and the original RGB three-channel were generated as the four-channel model input (**Figure 1**). Random image rotation, flipping (horizontal or vertical), image blur, random noise, gamma contrast variation, random translation, random scaling, inclination, and random discard pixels were used for data augmentation (11) (**Figure 2**).

The images chosen were randomly divided into training sets and test sets at a ratio of 8:2, and 5-fold cross-validation was used. We further developed another CAD-N for differentiating polyps with sizes ≤5 mm, and in building this model, the images chosen were randomly divided into training sets and test sets at a ratio of 9:1, and 10-fold cross-validation was used.

Hyperparameters were chosen through the AdamW optimizer. The model was trained for 300 epochs; the initial learning rate was 1e-4; cosine annealing was adopted as the attenuation method; and the final learning rate was 1e-6. When the training reached the preset loss value (loss <0.005) or 300 epochs, the training stopped. The output vector of the model was the probability (ranging from 0 to 100%) for each pathology type based on the evaluation of NBI images of the polyp/mucosa. The type with the highest probability was interpreted as the final diagnosis of the CAD-N. The CAD-N program was installed in a machine, which was named as “Endo Smart.” The video in the **Supplementary Material** shows how the CAD-N works.

To further validate the accuracy of the CAD-N, we collected 116 consecutive polyp videos (collected from the Sixth Affiliated Hospital, Sun Yat-sen University) that were recorded in the NBI model and confirmed with histopathology. The video recordings for validation were typically 10–20 s in length, and each video represented a polyp. The polyps in these videos had no overlap with the images in the training sets or the test sets. Then, 3 experts with more than 5 years of colonoscopy experience and 2 novices with 1 year of colonoscopy experience, all of whom were blinded to the histology results, were asked to classify the polyps in these videos according to NICE classifications.

### Statistical Analysis

Continuous variables for those with a normal distribution are presented as the mean (standard deviation, SD), and those without a normal distribution are presented as the median (interquartile range, IQR). Categorical variables are expressed as numbers (percentages). To evaluate the performance of the CAD-N for differentiating the pathology of polyps, the sensitivity, specificity, positive predictive value (PPV), negative predictive value (NPV), accuracy of each type of the CAD-N, and their 95% confidence intervals (CIs) were calculated. Moreover, a receiver operating characteristic curve (ROC) was plotted, and the area under the curve (AUC) was also calculated. The above calculation was performed by the Scikit-learn package in Python.

## Results

### Basic Information

A total of 10,573 images (7,032 images from 650 polyps and 3,541 normal mucous membrane images) from 478 patients were finally chosen for analysis, and the exact number of images used in each type is shown in **Table 1**. The mean age of the patients was 54.92 ( ± 13.56) years, and 295 patients (61.72%) were male. A total of 8,458 images were randomly divided into the training sets and 2,115 images into the test sets. The characteristics of the polyps/normal mucous membrane in the test sets are presented in **Table 2**.

**Table 1:** The number of the images selected in each type.

	Total	The training set	The test set
Type 1	2,496	1,983	513
Type 2	3,802	3,063	739
Type 3	734	591	143
Type 4	3,541	2,821	720

Type 1, hyperplastic or inflammatory polyps; type 2, adenomatous polyps, intramucosal cancer, or superficial submucosal invasive cancer; type 3, deep submucosal invasive cancer; and type 4, normal mucosa.

**Table 2:** The characteristics of the polyps/normal mucous membrane in the test sets.

Location	Type 1	Type 2	Type 3	Type 4
Rectum	41	13	26	30
Sigmoid colon	32	70	4	27
Descending colon	19	25	4	29
Splenic flexure	2	3	1	1
Transverse colon	22	49	2	22
Hepatic flexure	3	10	1	4
Ascending colon	7	30	3	21
Ileocecum	7	13	2	7
Location unclear	6	8	0	26
Total	139	221	43	167

Type 1, hyperplastic or inflammatory polyps; type 2, adenomatous polyps, intramucosal cancer, or superficial submucosal invasive cancer; type 3, deep submucosal invasive cancer; and type 4, normal mucosa.

### Image Analysis With a Deep Neural Network for All the Images

**Figure 3:**
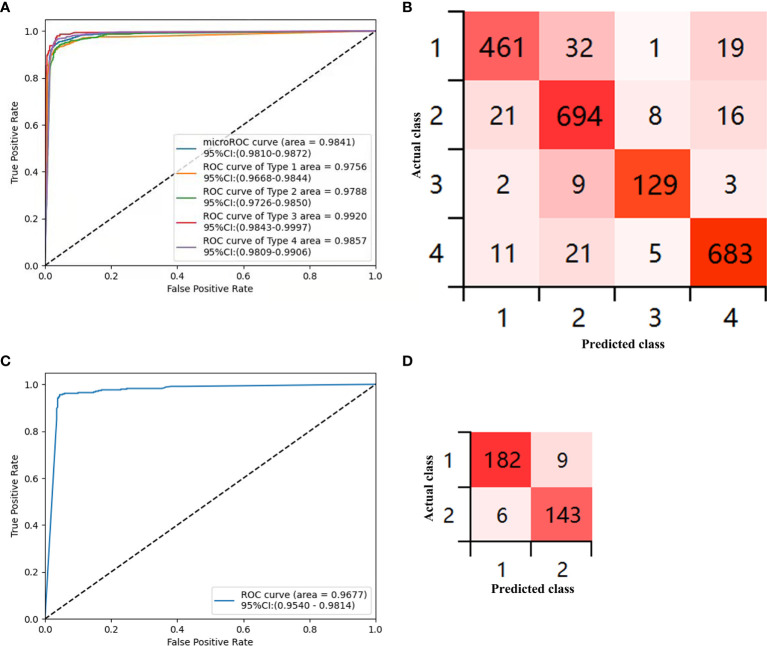
Diagnostic accuracy of the deep-learning model in the test sets. **(A)** Receiver operator curve (ROC) for the computer-aided detection (CAD)-N model for differentiation of each type versus other types and the overall micro-averaging ROC. **(B)** Confusion matrices show the pairwise comparison (number of images) in the test set. **(C)** ROC of type 1 versus type 2 for polyps ≤5 mm. **(D)** Confusion matrices for polyps ≤5 mm (type 1, hyperplastic or inflammatory polyps; type 2, adenomatous polyps, intramucosal cancer, or superficial submucosal invasive cancer; type 3, deep submucosal invasive cancer; and type 4, normal mucosa).

The sensitivity, specificity, PPV, NPV, and accuracy for each type of the CAD-N in the test sets were 89.86%, 97.88%, 93.13%, 96.79%, and 95.93% for type 1; 93.91%, 95.49%, 91.80%, 96.69%, and 94.94% for type 2; 90.21%, 99.29%, 90.21%, 99.29%, and 98.68% for type 3; and 94.86%, 97.28%, 94.73%, 97.35%, and 96.45% for type 4, respectively (**Table 3**). The overall accuracy was 93%. The ROC curves for each type versus other types and the overall micro-averaging ROC are shown in **Figure 3A**, and the confusion matrices showing the pairwise comparison (number of images) in the test sets are presented in **Figure 3B**.

**Table 3:** Diagnostic performance of the CAD-N for each type of polyps in the test sets by images.

	Type 1	Type 2	Type 3	Type 4	All
Sensitivity	89.86% (86.95%–92.19%)	93.91% (91.95%–95.42%)	90.21% (84.24%–94.08)	94.86% (93%–96.25%)	92.04% (90.50%–93.35%)
Specificity	97.88% (97.05%–98.48%)	95.49% (94.27%–96.47%)	99.29% (98.81%–99.58%)	97.28% (96.28%–98.01%)	94.86% (93%–96.25%)
PPV	93.13% (90.56%–95.04%)	91.8% (89.63%–93.55%)	90.21% (84.24%–94.08%)	94.73% (92.85%–96.14%)	97.2% (96.16%–97.96%)
NPV	96.79% (95.82%–97.54%)	96.69% (95.60%–97.52%)	99.29% (98.81–99.58%)	97.35% (96.36%–98.07)	86.02% (83.43%–88.26%)
Accuracy	95.93% (95.05%–96.70%)	94.94% (93.92%–95.80%)	98.68% (98.09%–99.08%)	96.45% (95.58%–97.16%)	93% (91.84%–94.01)

CAD, computer-aided detection; PPV, positive predictive value; NPV, negative predictive value.

Type 1, hyperplastic or inflammatory polyps; type 2, adenomatous polyps, intramucosal cancer, or superficial submucosal invasive cancer; type 3, deep submucosal invasive cancer; and type 4, normal mucosa.

### Image Analysis With a Deep Neural Network for Polyps ≤5 mm

We further analyzed polyps with sizes ≤5 mm, and 3,395 images were selected. The training sets contained 3,055 images from 376 polyps (220 were type 1 and 156 were type 2), and the test sets included 340 images from 158 polyps (88 were type 1 and 70 were type 2). The sensitivity, specificity, PPV, NPV, and accuracy for differentiating the type of polyps ≤5 mm in the test sets were 96.81% (93.21%–98.53%), 94.08% (89.13%–96.85%), 95% (91.29%–97.5%), 95.97% (91.49%–98.14%), and 95.59% (92.85%–97.31%), respectively. The ROC curves for differentiating the type of polyps ≤5 mm are shown in **Figure 3C**, and the confusion matrices for them are presented in **Figure 3D**.

### Validation Results of the Videos

Among the 116 videos (also 116 polyps) used for further validation, 65 polyps were type 1, and 51 polyps were type 2. The sensitivity, specificity, and accuracy of the CAD-N were 84.62% (73.94%–91.42%), 86.27% (73.13%–93.85%), and 85.34% (77.78%–90.64%), respectively. The diagnostic performance of the experts and novices seemed to not be significant different from that of the CAD-N (**Table 4**).

**Table 4:** Diagnostic performance of the CAD-N, experts, and novice in the validation videos.

	Sensitivity	Specificity	Accuracy
CAD-N	84.62% (73.94%–91.42%)	86.27% (73.13%–93.85%)	85.34% (77.78%–90.64%)
Expert 1	81.54% (70.45%–89.11%)	78.43% (65.37%–87.51%)	80.17% (72%–86.41%)
Expert 2	81.54% (70.45%–89.11%)	74.51% (61.13%–84.45%)	78.45% (70.12%–84.95%)
Expert 3	87.69% (77.55%–93.63%)	78.43% (65.37%–87.51%)	83.62% (75.83%–89.26%)
Novice 1	89.23% (79.40%–94.68%)	70.59% (57%–81.29%)	81.03% (72.95%–87.13%)
Novice 2	73.85% (62.05%–82.98%)	80.39% (67.54%–88.98%)	76.72% (68.25%–83.48%)

CAD, computer-aided detection.

## Discussion

Polypectomy of neoplastic polyps during colonoscopy remains the best policy for screening and prevention of colorectal cancer (14). Studies have shown that reliable real-time histopathology analysis is useful to improve the cost-effectiveness of colonoscopy in colorectal cancer surveillance and screening (15, 16). Except for the need to differentiate hyperplastic polyps and neoplastic polyps, ideally, endoscopists should be able to perceive the presence of invasive cancer during colonoscopy to make the suitable treatment decisions (17). Using white-light colonoscopy, the accuracy of real-time histopathology analysis was limited (18); the NICE classification is a simplified diagnostic criterion for the diagnosis of colorectal polyp histology and is widely used in centers without magnifying endoscopy (7, 8, 17). However, the judgment of the type may be somewhat subjective, and the confidence level of diagnosis may be different in endoscopists with different levels of experience (19, 20). Moreover, the detection of deep invasion of the polyps by non-magnifying NICE alone may not have sufficient accuracy compared with the magnifying endoscopic diagnosis (12). Hence, CAD-N, which enables the differentiation of real-time and objective histopathologic types of polyps even for non-magnifying images, is of great significance.

In this study, we used a DCNN-based machine learning algorithm to create the CAD-N model to help identify the histopathologic type of polyps in real time using NBI images, and the overall accuracy was excellent. Before our study, Rodriguez-Diaz et al. used 740 high-magnification NBI images from 607 polyps in 286 patients and over 65,000 subregions and developed a deep learning model to delineate polyp boundaries and to classify the histology of the polyps (11). In their study, they classified the polyps into two types, neoplastic and non-neoplastic, and the sensitivity, specificity, and NPV were 96% (92%–99%), 84% (74%–92%), and 91% (81%–97%), respectively. Their results were good, but they could not differentiate the neoplastic polyps with deep invasion.

Recently, Okamoto et al. used 4,156 NBI images to develop a CADx system using the NICE classification (12). Their model not only classified the neoplastic polyps and non-neoplastic polyps but also recognized the neoplastic polyps with deep invasion. In their study, the specificity for type 2 and the sensitivity for type 3 were not that satisfied. We think one of the reasons might be that in their training sets they used 3,670 magnifying images, and only 486 were non-magnifying images; if they used non-magnifying images in the test sets, the results would be influenced. Compared with their study, ours had some differences. First, the method they used to develop the model was ResNet, while we used ResNeSt, which is a new ResNet variant, preserving the overall ResNet structure without introducing additional computational costs and with better performance (13). Second, we collected more NBI images and built this model, whose test results were good, and the program was installed in a machine, which could give real-time histopathologic results during colonoscopy, as the video in the **Supplementary Material** shows.

We further validated the accuracy of the CAD-N using videos, as validation with videos rather than images could better reflect the clinical situation better. Although the accuracy in the validation videos was not as high as that in the test sets, it was close to the accuracy of the three experts with more than 5 years of colonoscopy experience (≥5,000 colonoscopies) and 2 novices with 1 year of colonoscopy experience (<5,000 colonoscopies). Byrne et al. also tested their models with videos, and their accuracy was 94%, sensitivity was 98%, and specificity was 83% (21). Actually, they used 125 polyp videos, but their model did not have enough confidence to predict the histology in 19 polyps, so they predicted the types of polyps in only 106 videos; hence, their accuracy, sensitivity, and specificity were overestimated and might be close to ours.

Several studies have focused on the classification of histopathologic types of diminutive polyps (<5 mm or ≤5 mm in size) (5, 11, 21). The ranges of the sensitivity, specificity, and NPV of their models were 95%–98%, 78.1%–88%, and 91.5%–97%, respectively. The Preservation and Incorporation of Valuable Endoscopic Innovations (PIVI) initiatives reported in the American Society of Gastrointestinal Endoscopy guidelines suggest that a histologic assessment of diminutive polyps should provide no less than 90% NPV for adenoma detection (22, 23). Their models all met the “leave *in situ*” criteria proposed by the PIVI initiatives. In Chen et al.’s study (5), they found that 3 of the novice endoscopists failed to meet the criteria, proving the advantages of the model over non-experts; moreover, their model could make the diagnosis faster than both the experts and non-experts, further suggesting the usefulness of the model. In our study, we also analyzed diminutive polyps, and our results met the criteria proposed by the PIVI initiatives. Therefore, we agreed that, with quick response and good reproducibility, CAD would become a very useful clinical tool especially for non-experts (24, 25).

Serrated lesions are another issue we are concerned about, ant the NICE system is not excellent in distinguishing sessile serrated adenoma/polyps (SSA/Ps) from benign hyperplastic polyps. To address this problem, the modified Sano (MS) classification and the Workgroup on Serrated Polyps and Polyposis (WASP) add-on classification have been developed (26, 27). Zorron et al. developed CAD for characterization of colorectal lesions, including the differentiation of serrated lesions (28), and the AUC for the SSA was 0.85 in the validation sets. In previous studies, the number of SSA/Ps was limited (11, 21), and in our study, we collected only 144 images of SSAs, which is also a very small number and a limitation of our study. We think that, in the future, if we want to develop better convincible CADx, we should include more images of SSA/Ps, especially for the diminutive images, to meet the “resect and discard” strategy (23).

One of the strengths of this study is the relatively large number of images in the training sets, and the diagnostic performance of CAD-N in the test sets was good, with real-time histopathologic diagnosis. However, there are some limitations in this study. First, there was a lack of external validation, so we do not know how the CAD-N worked in other centers. In the future, we hope we can do some research to answer this question. Second, as mentioned above, the number of images of SSA/Ps included in this study was limited, and we should accumulate more images of SSA/Ps in the next version. Third, there were no type 3 polyps in the validation videos, and the diagnostic performance of the CAD-N using the validation videos was not as excellent as that in the test sets; hence, perhaps more polyps were needed to improve the models.

In conclusion, we developed a real-time AI-based histologic classification model for colorectal polyps using NBI images, which could not only accurately distinguish hyperplastic polyps and neoplastic polyps but also differentiate the neoplastic polyps with deep invasion, helping clinical management and documentation of optical histology results. Future work is required as more images are required, especially of SSA/Ps, for further development and in designing larger external validation studies. Although the CAD-N has good performance, we believe it still cannot replace the role of endoscopists, as the latter must drive the scope stably, clean away debris and foam, observe the mucosal surfaces without missing areas, and ensure clear visualization of the lesions to let the CAD-N focus on the lesion and make a diagnosis. Moreover, endoscopists can apply their knowledge and skills during intervention. Deep machine learning technology will continually learn from us, helping us to improve our performance.

## Data Availability Statement

The raw data supporting the conclusions of this article will be made available by the authors, without undue reservation.

## Ethics Statement

This study was approved by the Institutional Review Board of the Sixth Affiliated Hospital, Sun Yat-sen University (Approval Code: 2021ZSLYEC-221), and Guangdong Second Provincial Central Hospital (Approval Code: 2021-KZ-228-02), and it has been registered in the Chinese Clinical Trial Registry (No. ChiCTR2100051800). Written informed consent from the participants’ legal guardian/next of kin was not required to participate in this study in accordance with the national legislation and the institutional requirements.

## Author Contributions

YLu and JW designed the study and wrote the manuscript. YLu, JW, XZ, MH, YC, and JS collected the clinical data and the images. YLuo, YF and their teams processed the images and built the models. YLu, JW, YLuo, and YF performed the statistical analysis. YLu, XZ, MH, YC, and JS performed the video validation. MZ, CL, and JS revised the manuscript. All authors contributed to the article and approved the submitted version.

## Funding

This study was funded by grants from the National Key R&D Program of China (Grant No. 2017YFC1308800) and the Sixth Affiliated Hospital of Sun Yat-sen University of Horizontal Program (Grant No. H202101162024041054).

## Conflict of Interest

The authors declare that the research was conducted in the absence of any commercial or financial relationships that could be construed as a potential conflict of interest.

## Publisher’s Note

All claims expressed in this article are solely those of the authors and do not necessarily represent those of their affiliated organizations, or those of the publisher, the editors and the reviewers. Any product that may be evaluated in this article, or claim that may be made by its manufacturer, is not guaranteed or endorsed by the publisher.
